# Supplementary figures and images for: Lung epithelial cells have virus-specific and shared gene expression responses to infection by diverse respiratory viruses
Source: PLoS One. 2017 Jun 2;12(6):e0178408. doi: 10.1371/journal.pone.0178408 (PMC5456070; doi:10.1371/journal.pone.0178408)

MHV

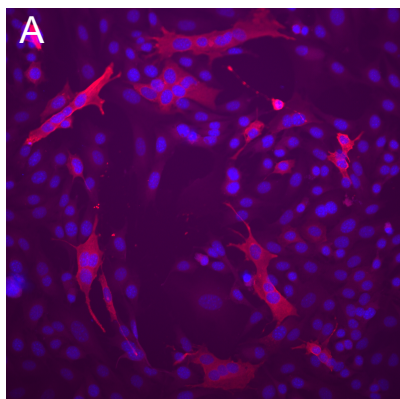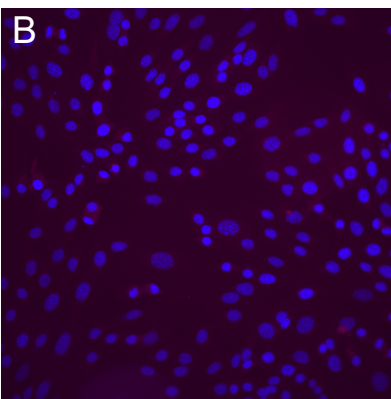

RV

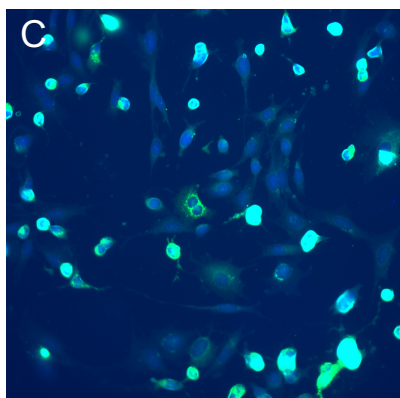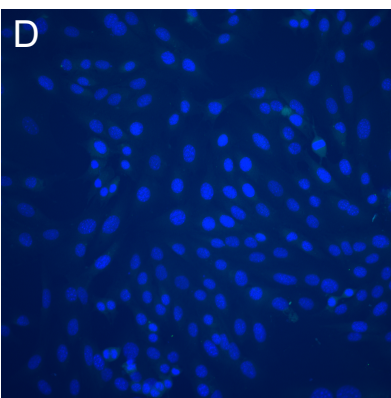

PR8

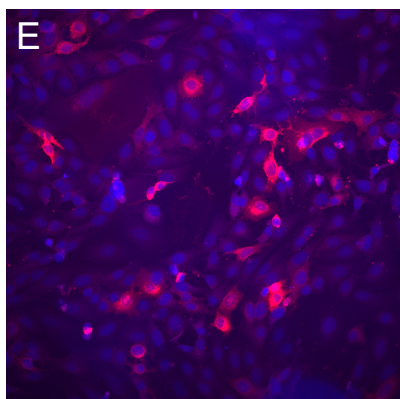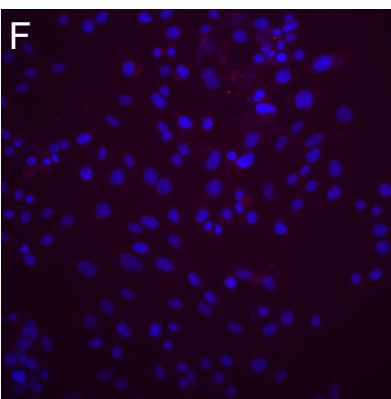

G

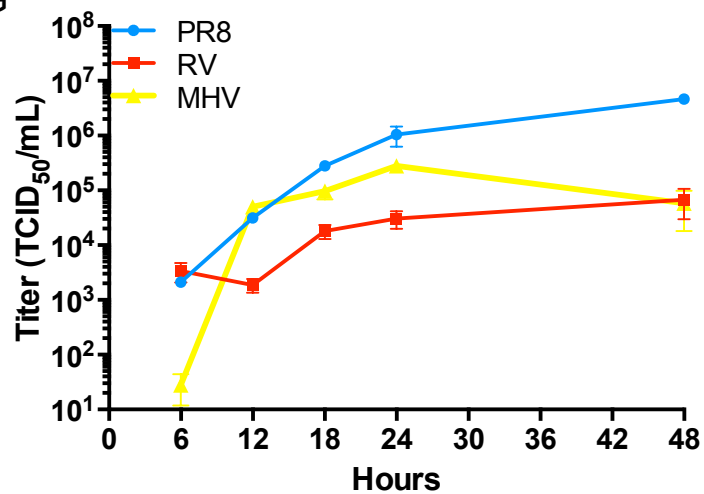

Supplement: S1 Fig — LA4 cells were inoculated with (A) 3 PFU/cell MHV, (C) 3 TCID50/cell RV, or (E) 1 PFU/cell PR8, or were mock inoculated (B, D, F). Cells were fixed in 4% formaldehyde and permeabilized with Triton X100. (A, B) MHV infection was evaluated using a monoclonal antibody that recognizes the nucleocapsid protein (provided by Dr. Julian Leibowitz, Texas A&M University), followed by goat anti-mouse-555 (Invitrogen). (C, D) RV antigens were detected using RV1B antiserum (ATCC) and goat anti-guinea pig-488 (Rockland, Gilbertsville, PA). Goat antiserum NR-3148, which recognizes the hemagglutinin protein of PR8 (BEI Resources), and anti-goat-555 (Invitrogen) were used to detect PR8 infection. Nuclei were stained with DAPI and were photographed on a Nikon Eclipse Ti Epifluorescent Microscope with a Nikon DS-Qi2 camera and NIS Elements software (Nikon). (G) LA4 cells were inoculated with MHV, RV, or PR8, as described above and viral titers in the supernatant medium was analyzed by TCID50 assays in MDCK (PR8), HeLa (RV), and 17cl1 (MHV) cell lines. Titers are the average and SEM of four replicate samples at each time point. (PDF) [file pone.0178408.s001.pdf]
