# Supplementary material for: Lung epithelial cells have virus-specific and shared gene expression responses to infection by diverse respiratory viruses
Source: PLoS One. 2017 Jun 2;12(6):e0178408. doi: 10.1371/journal.pone.0178408 (PMC5456070; doi:10.1371/journal.pone.0178408)

Gene Symbol	Gene Full Name	Virus	Array Log2 Fold Change at 24 h	ΔΔCt at 24 h
Bst2	bone marrow stromal cell antigen 2	MHV-1	0.8459	0.6657
Cxcl10	chemokine (C-X-C motif) ligand 10	MHV-1	4.3212	5.3203
Oas1a	2'-5' oligoadenylate synthetase 1A	MHV-1	1.2753	1.2028
Tnf	tumor necrosis factor	MHV-1	3.1622	4.9143
Bst2	bone marrow stromal cell antigen 2	PR8	6.1580	7.0620
Cxcl10	chemokine (C-X-C motif) ligand 10	PR8	7.1476	8.0997
Icam1	intercellular adhesion molecule	PR8	1.8706	2.3305
Oas1a	2'-5' oligoadenylate synthetase 1A	PR8	6.5127	8.1082
Bst2	bone marrow stromal cell antigen 2	RV1B	4.5160	3.7276
Cxcl10	chemokine (C-X-C motif) ligand 10	RV1B	5.2792	4.3365
Oas1a	2'-5' oligoadenylate synthetase 1A	RV1B	4.5920	4.3453
Tnf	tumor necrosis factor	RV1B	0.0391	1.4836
Icam1*	intercellular adhesion molecule	MHV-1	3.3227	-1.3873
Tnf*	tumor necrosis factor	PR8	4.2388	9.0176
Icam1*	intercellular adhesion molecule	RV1B	6.1080	-0.7722

*outliers with Cook's distance > 0.5 were removed from linear regression

	qPCR primer sequences
Tnf	5' – CCAAAGGGATGAGAAGTTCC – 3'
	5' – CTCCACTTGGTGGTTTGC – 3'
Cxcl10	5' – GGATGGCTGTCCTAGCTCTG – 3'
	5' – TGAGCTAGGGAGGACAAGG – 3'
Icam1	5' – CGCTGTGCTTTGAGAACTG – 3'
	5' – GGTGAGGTCCTTGCCTAC – 3'
Bst2	5' – TCAGGAGTCCCTGGAGAAG – 3'
	5' – ATGGAGCTGCCAGAGTCCAC – 3'
Oas1a	5' – CTTTGATGTCCTGGGTCATG – 3'
	5' – CTCCGTGAAGCAGGTAGAG – 3'
β-Actin	5' – AAGTCCCTCACCCTCCCAAAAG – 3'
	5' – AAGCAATGCTGTCACCTTCCC – 3'

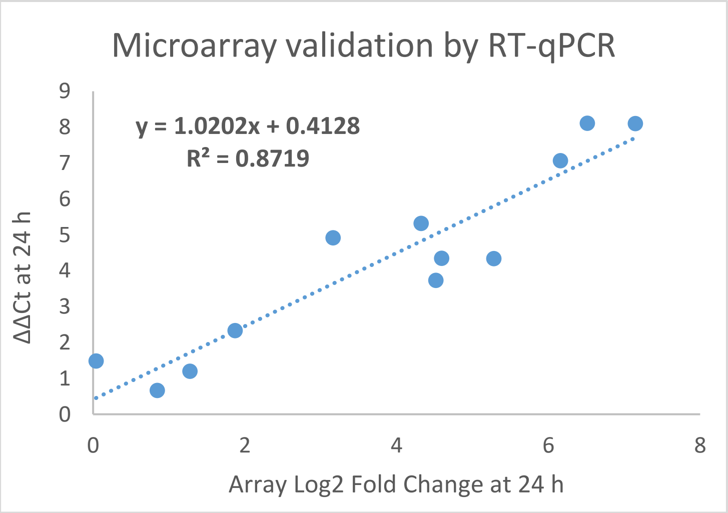

Supplement: S2 Fig — LA4 cells were inoculated with virus using the same MOI’s as for the microarray study and RNA was extracted at 24 h post-infection using Trizol (Ambion). RNA was converted to cDNA using random hexamers and SuperScript VILO (Invitrogen). Five genes with differential expression by microarray analysis were validated by qPCR analysis using SYBR green (PowerUP, Applied Biosystems) and the primer pairs listed in the figure on a StepOne Plus Instrument (Applied Biosystems). CT values from triplicate qPCR reactions were averaged and normalized to β-actin before calculating the fold change values of virus-infected samples vs. mock. Linear regression was used to compare fold change values between qPCR and microarray with removal of outliers (*) with Cook's distance > 0.5. (PDF) [file pone.0178408.s002.pdf]
