# Supplementary material for: Lung epithelial cells have virus-specific and shared gene expression responses to infection by diverse respiratory viruses
Source: PLoS One. 2017 Jun 2;12(6):e0178408. doi: 10.1371/journal.pone.0178408 (PMC5456070; doi:10.1371/journal.pone.0178408)
Supplement: S3 Fig — Genes with significantly up-regulated expression compared to mock at 24 h (see Fig 2) were used to query the Interferome v.2.01 database. Interferon-regulated genes were divided into functional groups and heat maps were generated using log2-fold change values for each virus at 24 h compared to mock-inoculated controls. Heat maps of additional functional groups can be found in Fig 4. Gene names are indicated to the right of each row and statistically significant values are outlined in black. (PDF) [file pone.0178408.s003.pdf]

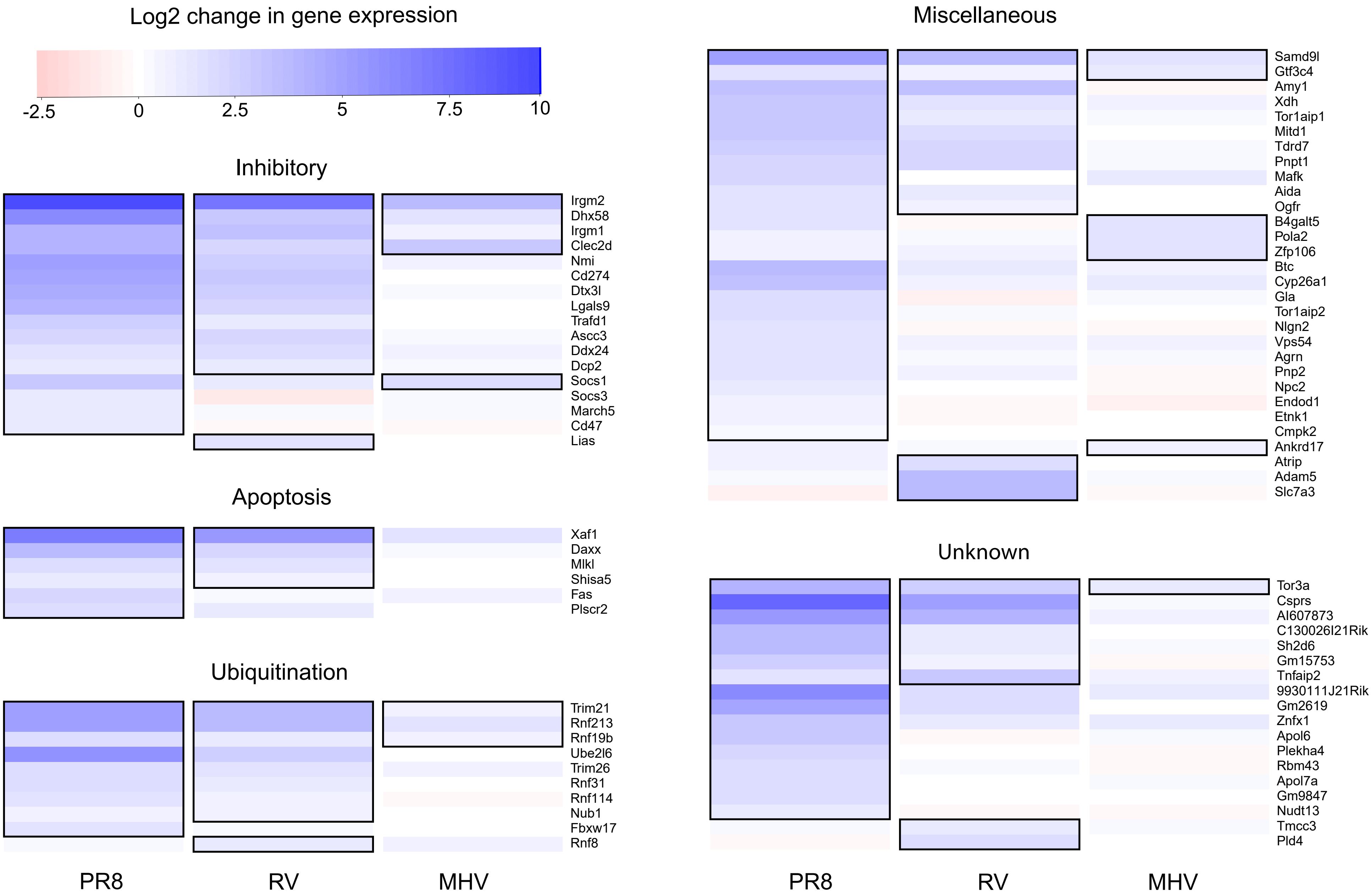
Log2 change in gene expression
-2.5
0
2.5
5
7.5
10
Inhibitory
Irgm2
Dhx58
Irgm1
Clec2d
Nmi
Cd274
Dtx3l
Lgals9
Trafd1
Ascc3
Ddx24
Dcp2
Socs1
Socs3
March5
Cd47
Lias
Apoptosis
Xaf1
Daxx
Mlkl
Shisa5
Fas
Plscr2
Ubiquitination
Trim21
Rnf213
Rnf19b
Ube2l6
Trim26
Rnf31
Rnf114
Nub1
Fbxw17
Rnf8
Miscellaneous
Samd9l
Gtf3c4
Amy1
Xdh
Tor1aip1
Mitd1
Tdrd7
Pnpt1
Mafk
Aida
Ogfr
B4galt5
Pola2
Zfp106
Btc
Cyp26a1
Gla
Tor1aip2
Nlgn2
Vps54
Agrn
Pnp2
Npc2
Endod1
Etnk1
Cmpk2
Ankrd17
Atrip
Adam5
Slc7a3
Unknown
Tor3a
Csprs
AI607873
C130026I21Rik
Sh2d6
Gm15753
Tnfaip2
9930111J21Rik
Gm2619
Znfx1
Apol6
Plekha4
Rbm43
Apol7a
Gm9847
Nudt13
Tmcc3
Pld4
PR8
RV
MHV
PR8
RV
MHV
